# Supplementary material for: Co-Clinical Imaging Metadata Information (CIMI) for Cancer Research to Promote Open Science, Standardization, and Reproducibility in Preclinical Imaging
Source: Tomography. 2023 May 11;9(3):995–1009. doi: 10.3390/tomography9030081 (PMC10204428; doi:10.3390/tomography9030081)
Supplement: Supplementary file 1 [file tomography-09-00081-s001.zip › tomography-2209289-supplementary.pdf]

Supplemental Tables

**Table S1:** Summary results for Imaging Common category			
**Element**	**Assessment**	**DICOM Tag**	**Definition**
These values not found in DICOM images			
Rodent temperature during imaging	Essential	NA	Subject temperature and the means for taking the temperature can be expressed in a DICOM Structured Report. One would not expect to find these two items in a DICOM image.
Route temperature was assessed	Essential	NA	Source of temperature reading (for example, rectal).
General Series Module			
Series Description	Essential	0008,103E	Description of the Series.
General Acquisition Module			
Acquisition Date	Essential	0008,0022	The date the acquisition of data that resulted in this instance started.
Acquisition Time	Essential	0008,0032	The time the acquisition of data that resulted in this instance started.
General Series Module			
Body Part Examined	Essential	0018,0015	Text description of the part of the body examined. *DICOM has defined terms for this field*
Patient Position	Essential	0018,5100	Patient position descriptor relative to the patient support device. *Uses defined terms such as HFP (Head First-Prone), HFS (Head First-Supine), …*
General Equipment Module (PET Image, MR Image, Enhanced MR Image, CT Image, Enhanced CT Image)			
Manufacturer	Essential	0008,0070	Manufacturer of the equipment that produced the Composite Instances.
Station Name	Recommended	0008,1010	User defined name identifying the machine locally that produced the Composite Instances.
Manufacturer's Model Name	Essential	0008,1090	Manufacturer's model name of the equipment that produced the Composite Instances.
Device Serial Number	Recommended	0018,1000	Manufacturer's serial number of the equipment that produced the Composite Instances.

Software Versions	Essential	0018,1020	Manufacturer's designation of software version of the equipment that produced the Composite Instances.
General Image Module (PET Image, MR Image, CT Image)			
Patient Orientation	Essential	0020,0020	Patient direction of the rows and columns of the image.
Anatomical Orientation Type	Essential	0010,2210	The anatomical orientation type used in Instances generated by this equipment.<br><br>Enumerated Values: BIPED, QUADRUPED
Image Plane Module (PET Image, MR Image, CT Image)			
Pixel Spacing	Essential	0028,0030	Physical in-plane distance in the patient between the center of each pixel, specified by a numeric pair - adjacent row spacing (delimiter) adjacent column spacing in mm.
Image Orientation (Patient)	Essential	0020,0037	The direction cosines of the first row and the first column with respect to the patient.
Image Position (Patient)	Essential	0020,0032	The x, y, and z coordinates of the upper left hand corner (center of the first voxel transmitted) of the image, in mm.
Slice Thickness	Essential	0018,0050	Nominal slice thickness, in mm.
Spacing Between Slices	Essential	0018,0088	Spacing between adjacent slices, in mm. The spacing is measured from the center-to-center of each slice.
Slice Location	Recommended	0020,1041	Relative position of the image plane expressed in mm.
Voxel Size	Essential	NA	*Computed from Slice Thickness and Pixel Spacing*
Image Pixel Module (PET Image, MR Image, Enhanced MR Image, CT Image, Enhanced CT Image)			
Rows	Recommended	0028,0010	Number of rows in the image.
Columns	Recommended	0028,0011	Number of columns in the image.
Contrast/Bolus Module (MR Image, CT Image)			
Contrast/Bolus Agent	Essential	0018,0010	Contrast or bolus agent.
Contrast/Bolus Route	Essential	0018,1040	Administration route of contrast agent.
Contrast/Bolus Administrative Route Sequence ->	Recommended	0018,0014 > 0018,002A	Sequence that identifies any additional drug that is administered with the

Additional Drug Sequence			contrast agent bolus. One or more Items are permitted in this Sequence.
Contrast/Bolus Volume	Essential	0018,1041	Volume injected in milliliters of diluted contrast agent.
Contrast/Bolus Start Time	Essential	0018,1042	Time of start of injection.
Contrast/Bolus Stop Time	Essential	0018,1043	Time of end of contrast injection.
Contrast/Bolus Total Dose	Essential	0018,1044	Total amount in milliliters of the undiluted contrast agent.
Contrast Flow Rate	Essential	0018,1046	Rate(s) of injection(s) in milliliters/sec.
Contrast Flow Duration	Essential	0018,1047	Duration(s) of injection(s) in seconds. Each Contrast Flow Duration value shall correspond to a value of Contrast Flow Rate (0018,1046).
Contrast/Bolus Ingredient	Essential	0018,1048	Active ingredient of agent. Defined Terms: IODINE, GADOLINIUM, CARBON DIOXIDE, BARIUM
Contrast/Bolus Ingredient Concentration	Essential	0018,1049	Milligrams of active ingredient per milliliter of (diluted) agent.

Table S2: Summary results for PET & SPECT Imaging category

**Element**	**Assessment**	**DICOM Tag**	**Definition**
PET Series Module			
Units	Essential	0054,1001	Pixel value units. The units of the pixel values obtained after conversion from the stored pixel values (SV) (Pixel Data (7FE0,0010)) to pixel value units (U), as defined by Rescale Intercept (0028,1052) and Rescale Slope (0028,1053). *Defined terms include CNTS, NONE, CM2, CM2ML, PCT, CPS, BQML, …*
SUV type	Essential	0054,1006	Type of Standardized Uptake Value (SUV). *Enumerated values: BSA, BW, LBM, LBMJAMES128, LBMJANMA, IBW*
Counts Source	Essential	0054,1002	The primary source of counts. The primary source leads to the underlying image Units (0054,1001), as opposed to

			secondary sources that are used during reconstruction correction.<br>Enumerated Values: EMISSION, TRANSMISSION
Series Type	Essential	0054,1000	A multi-valued indicator of the type of Series. *Value 1: STATIC, DYNAMIC, GATED, WHOLE BODY.*<br>*Value 2: IMAGE, REPROJECTION*
Reprojection method	Recommended	0054,1004	Method for projecting volumetric data onto planar projection. Required if Series Type (0054,1000), Value 2 is REPROJECTION.<br>Defined Terms: SUM, MAX PIXEL
Number of Time Slots	Essential	0054,0071	The maximum number of Time Slots that may exist in this Series. Required if Series Type (0054,1000), Value 1 is GATED.
Number of Time slices	Essential	0054,0101	The maximum number of Time Slices that may exist in this Series. Required if Series Type (0054,1000), Value 1 is DYNAMIC.
Number of Slices	Essential	0054,0081	The maximum number of Slices that may exist in this Series.
Corrected Image	Essential	0028,0051	One or more values that indicate which, if any, corrections have been applied to the image.
Randoms Correction Method	Essential	0054,1100	Type of randoms correction processing. Defined Terms: NONE (no randoms correction), DLYD (delayed event subtraction), SING (singles estimation)
Attenuation Correction Method	Essential	0054,1101	A textual description of the attenuation correction processing. e.g., measured vs. calculated, transmission source type (ring, line, point), assumed patient geometry (polygon, ellipse, segmented, attenuation coefficient, skull thickness), post-injection transmission, smoothing.
Scatter Correction Method	Essential	0054,1105	A textual description of the scatter correction processing. e.g., convolution-subtraction, dual energy window, model-based, use of attenuation data.

Decay Correction	Essential	0054,1102	The real-world event to which images in this Series were decay corrected. See Section C.8.9.1.1.5 for explanation.<br>Defined Terms: NONE, START, ADMIN
Reconstruction method	Essential	0054,1103	Textual description of reconstruction processing, e.g., 2D filtered backprojection, 2D iterative, 3D PROMIS, 3D FAVOR, 3D iterative.
Convolution Kernel	Essential	0018,1210	Textual description of the convolution kernel(s) used to reconstruct the data (e.g., name, cutoff, radial/axial/angular, mathematical form, DC handling).
Field of View Dimensions	Essential	0018,1149	Dimensions of the field of view, in mm. Transverse detector diameter followed by axial width.
Collimator Type	Recommended	0018,1181	Collimator Type.<br>Defined Terms: NONE, RING
Collimator / Grid Name	Recommended	0018,1180	Label describing the collimator used (LEAP, hires, etc.).
Coincidence Window Width	Recommended	0054,1210	The width of the coincidence timing window, in nsec. The maximum time difference accepted between two single events.
Type of Detector Motion	Recommended	0054,0202	Describes the detector motion during acquisition.<br>Defined Terms: NONE, STEP AND SHOOT, CONTINUOUS, WOBBLE, CLAMSHELL
PET Isotope Module			
Radionuclide Code Sequence	Essential	0054,0016 > 0054,0300	*A coded item in a DICOM image that identifies the radionuclide. The coded item has several components and is not a simple text item.*
Radiopharmaceutical Route	Essential	0054,0016 > 0018,1070	Route of administration.
Radiopharmaceutical Volume	Recommended	0054,0016 > 0018,1071	Volume of administered radiopharmaceutical in cubic cm.

Radiopharmaceutical Start DateTime	Essential	0054,0016 > 0018,1078	Date and time of start of administration. The actual date and time of radiopharmaceutical administration to the patient for imaging purposes, using the same time base as Series Time (0008,0031).
Radionuclide Total Dose	Essential	0054,0016 > 0018,1074	The radiopharmaceutical dose administered to the patient measured in Becquerels (Bq) at the Radiopharmaceutical Start Time (0018,1072).
Radiopharmaceutical Specific Activity	Recommended	0054,0016 > 0018,1077	The activity per unit mass of the radiopharmaceutical, in Bq/micromole, at the Radiopharmaceutical Start Time (0018,1072).
Radiopharmaceutical	Essential	0054,0016 > 0018,0031	Name of the radiopharmaceutical.
Intervention Drug Name	Essential	0018,0026 > 0018,0034	Name of the intervention drug. *Part of Intervention Drug Information Sequence that describes intervention drugs used.*
Solvent	Essential		Solvent used to prepare imaging agent for injection.
PET Multi-gated Acquisition Module			
Trigger or Source Type	Recommended	0018,1061	Text indicating trigger source. Defined Terms: EKG
Heart rate	Recommended	0018,1088	Average number of heart beats per minute for the collection period for this image. This shall include all accepted beats as well as rejected beats.
NM/PET Patient Orientation Module			
Patient Gantry Relationship Code Sequence	Essential	0054,0414	Describes the orientation of the patient with respect to the gantry. *Uses a defined vocabulary where the coded values will map to terms such as oblique, headfirst, feet-first, transverse …*
General Image Module			
Lossy Image Compression	Essential	0028,2110	Specifies whether the Source Images have undergone lossy compression (at a point in their lifetime).

PET Image Module			
Image Type	Essential	0008,0008	*One DICOM element with multiple fields that will indicate if the image is based on original/source data or is derived from other data; if the image was created as a direct result of the patient examination or after the original examination*
Frame time	Essential	0018,1063	Nominal duration per individual frame, in msec. Required if Series Type (0054,1000), Value 1 is GATED.
Image Index	Essential	0054,1330	An index identifying the position of this image within a PET Series. See
Actual Frame Duration	Essential	0018,1242	Elapsed time of data acquisition in msec per each frame.
Decay Factor	Essential	0054,1321	The decay factor that was used to scale this image. Required if Decay Correction (0054, 1102) is other than NONE. If decay correction is applied, all images in the Series shall be decay corrected to the same time.
Dose Calibration Factor	Essential	0054,1322	Factor that was used to scale this image from counts/sec to Bq/ml using a dose calibrator. The value shall be one if no dose calibration was applied. See Section C.8.9.4.1.8 for explanation.
Isocenter Position	Recommended	300A,012C	Isocenter coordinates (x,y,z), in mm. Specifies the location of the machine isocenter in the Patient-Based Coordinate System associated with the Frame of Reference. It allows transformation from the Equipment-Based Coordinate System to the Patient-Based Coordinate System.
Iterative Reconstruction Method	Essential	0018,9769	Iterative Reconstruction Method used. (yes/no)
Iterative reconstruction number	Essential	0018,9739	Required if Frame Type (0008,9007) Value 1 equals ORIGINAL and Iterative Reconstruction Method (0018,9769) equals YES. May be present otherwise.

Iterative Reconstruction number of subsets	Essential	0018,9740	Required if Frame Type (0008,9007) Value 1 equals ORIGINAL and Iterative Reconstruction Method (0018,9769) equals YES. May be present otherwise.
Number of views (SPECT)	Recommended	0054,0053	Number of angular views in this rotation.

**Table S3:** Summary results for MR Imaging category			
**Element**	**Assessment**	**DICOM Tag**	**Definition**
MR Image Module (MR Image)			
Image Type	Essential	0008,0008	*For MR data, the 3rd value has these defined terms: DENSITY MAP, DIFFUSION MAP, IMAGE ADDITION, MODULUS SUBTRACT, MPR, OTHER, PHASE MAP, PHASE SUBTRAT, PROJECTION IMAGE, T1 MAP, T2 MAP, VELOCITY MAP*
Photometric Interpretation	Essential	0028,0004	Specifies the intended interpretation of the pixel data. *Enumerated values: MONOCHOROME1, MONOCHROME2*
Scanning Sequence	Essential	0018,0020	Description of the type of data taken. Enumerated Values: SE (Spin Echo), IR (Inversion Recovery), GR (Gradient Recalled), EP (Echo Planar), RM (Research Mode)
Sequence Variant	Essential	0018,0021	Variant of the Scanning Sequence. Defined Terms: SK (segmented k-space), MTC (magnetization transfer contrast), SS (steady state), TRSS (time reversed steady state), SP (spoiled), MP (MAG prepared), OSP (oversampling phase), NONE (no sequence variant)
Scan Options	Essential	0018,0022	Parameters of scanning sequence. Defined Terms: PER, RG, CG, PPG, RC, PFF, PFP, SP, FS
Respiratory Gating	Essential	NA	Determined from Scan Options, 0018,0022

MR Acquisition Type	Essential	0018,0023	Identification of data encoding scheme.<br>Enumerated Values: 2D (frequency x phase), 3D (frequency x phase x phase)
Repetition Time	Essential	0018,0080	The period of time in msec between the beginning of a pulse sequence and the beginning of the succeeding (essentially identical) pulse sequence.
Echo Time	Essential	0018,0081	Time in ms between the middle of the excitation pulse and the peak of the echo produced (kx=0). In the case of segmented k-space, the TE(eff) is the time between the middle of the excitation pulse to the peak of the echo that is used to cover the center of k-space (i.e., -kx=0, ky=0).
Echo Train Length	Essential	0018,0091	Number of lines in k-space acquired per excitation per image.
Inversion Time	Essential	0018,0082	Time in msec after the middle of inverting RF pulse to middle of excitation pulse to detect the amount of longitudinal magnetization.
Trigger Time	Essential	0018,1060	Time, in msec, between peak of the R wave and the peak of the echo produced.
Sequence Name	Essential	0018,0024	User defined name for the combination of Scanning Sequence (0018,0020) and Sequence Variant (0018,0021).
Angio Flag	Essential	0018,0025	Angio Image Indicator. Primary image for Angio processing.<br>Enumerated Values: Y (Image is Angio), N (Image is not Angio)
Number of averages	Essential	0018,0083	Number of times a given pulse sequence is repeated and combined with the same parameter values.
Imaging Frequency	Essential	0018,0084	Precession frequency in MHz of the nucleus being addressed.

Imaged Nucleus	Essential	0018,0085	Nucleus that is resonant at the imaging frequency. Examples: 31P, 1H.
Echo Number(s)	Essential	0018,0086	The echo number used in generating this image. In the case of segmented k-space, it is the effective Echo Number.
Magnetic Field Strength	Essential	0018,0087	Nominal field strength of MR magnet, in Tesla.
Number of Phase Encoding Steps	Essential	0018,0089	Total number of lines in k-space in the 'y' direction collected during acquisition.
Percent Sampling	Essential	0018,0093	Fraction of acquisition matrix lines acquired in the first phase encoding direction, expressed as a percent.
Percent Phase Field of View	Essential	0018,0094	Ratio of field of view dimension in phase direction to field of view dimension in frequency direction, expressed as a percent.
Pixel Bandwidth	Essential	0018,0095	Reciprocal of the total sampling period, in hertz per pixel.
Heart Rate	Essential	0018,1088	Average number of heart beats per minute for the collection period for this image. This shall include all accepted beats as well as rejected beats.
Receive Coil Name	Essential	0018,1250	Receive coil used.
Coil Type and Diameter	Essential	NA	*Derived from Receive Coil Name*
Transmit Coil Name	Essential	0018,1251	Transmit coil used.
Acquisition Matrix	Essential	0018,1310	Dimensions of the acquired frequency /phase data before reconstruction.<br>Multi-valued: frequency rows\frequency columns\phase rows\phase columns.
In-plane Phase Encoding Direction	Essential	0018,1312	The axis of phase encoding with respect to the image.<br>Enumerated Values: ROW (phase encoded in rows.), COL (phase encoded in columns.)

Flip Angle	Essential	0018,1314	Steady state angle in degrees to which the magnetic vector is flipped from the magnetic vector of the primary field.
SAR	Recommended	0018,1316	Calculated whole body Specific Absorption Rate in watts/kilogram.
Variable Flip Angle Flag	Essential	0018,1315	Flip angle variation applied during image acquisition.<br>Enumerated Values: Y, N
dB/dt	Recommended	0018,1318	The rate of change of the gradient coil magnetic flux density with time (T/s).
Temporal Position Identifier	Essential	0020,0100	Temporal order of a dynamic or functional set of Images.
Number of temporal positions	Essential	0020,0105	Total number of temporal positions prescribed.
Temporal Resolution	Essential	0020,0110	Time delta between Images in a dynamic or functional set of Images.
Isocenter Position	Recommended	300A,012C	Isocenter coordinates (x,y,z), in mm. Specifies the location of the machine isocenter in the Patient-Based Coordinate System associated with the Frame of Reference. It allows transformation from the Equipment-Based Coordinate System to the Patient-Based Coordinate System.
B1rms	Recommended	0018,1320	B1+ rms value in units of microtesla (µT) for the acquisition producing the image. See [IEC 60601-2-33].
Number of slices	Essential	NA	*Calculated from the image data itself*
Acquired Voxel Size	Essential	NA	*Calculated from Acquisition Matrix, Rows, Columns, Pixel Spacing (x,y), Slice Thickness (z)*
Enhanced MR Image Module (Enhanced MR Image)			
Acquisition Duration	Essential	0018,9073	The time in seconds needed to complete the acquisition of data.
Resonant Nucleus	Essential	0018,9100	Nucleus that is resonant at the transmitter frequency.<br><br>Defined Terms: 1H, 3HE, 7LI, 13C, 19F, 23NA, 31P, 129XE

k-space Filtering	Essential	0018,9064	Describes k-space filtering applied. Shall be NONE if no k-space filter. Defined Terms: COSINE, COSINE_SQUARED, FERMI, GAUSSIAN, HAMMING, HANNING, LORENTZIAN, LRNTZ_GSS_TRNSFM, RIESZ, TUKEY, NONE
View Code Sequence	Essential	0054,0220	Sequence that describes the projection of the anatomic region of interest.
Slice Progression Direction	Essential	0054,0500	Describes the anatomical direction in which a set of slices is progressing (see Section 10.20.1.1).<br>*Uses enumerated values.*
Acquisition Contrast	Essential	0008,9209	Indication of acquisition contrast used with frames in the SOP Instance.<br>Defined Terms: DIFFUSION, FLOW_ENCODED, FLUID_ATTENUATED, PERFUSION, PROTON_DENSITY, STIR, TAGGING, T1, T2, T2_STAR, TOF, UNKNOWN, MIXED
Enhanced MR Image Functional Group Macros: Pixel Value Transformation			
Rescale Intercept	Essential	5200,9229 > 0028,9145 > 0028,1052	The value b in relationship between stored values (SV) and the output units.<br>Output units = m*SV+b
Rescale Slope	Essential	5200,9229 > 0028,9145 > 0028,1053	The slope (m) in the equation specified in Rescale Intercept (0028,1052).
Enhanced MR Image Functional Group Macros: MR Diffusion			
Diffusion b-value	Essential	5200,9229 > 0018,9117 > 0018,9087	Identifies the diffusion parameters of this frame.<br><br>Diffusion sensitization factor in sec/mm2. This is the actual b-value for original frames and those derived from frames with the same b-value, or the most representative b-value

			when derived from images with different b-values.
Diffusion Directionality	Essential	5200,9229 > 0018,9117 > 0018,9075	Identifies the diffusion parameters of this frame.<br><br>Specifies whether diffusion conditions for the frame are directional, or isotropic with respect to direction. Defined Terms: DIRECTIONAL, BMATRIX, ISOTROPIC, NONE
Diffusion b-matrix Sequence	Essential	5200,9229 > 0018,9117 > 0018,9601	The directional diffusion sensitization expressed as a 3x3 matrix with diagonal symmetry (with six unique elements from which the other elements can be derived).<br>The rows and columns of the matrix are the X (right to left), Y (anterior to posterior) and Z (foot to head) patient-relative orthogonal axes as defined in Section C.7.6.2.1.1.<br>The values are in units of ms/mm2.
Enhanced MR Image Functional Group Macros: Respiratory Synchronization			
Respiratory Motion Compensation Technique	Essential	5200,9229 > 0018,9170	Applied technique to reduce respiratory motion artifacts.<br><br>Defined Terms: NONE, BREATH_HOLD, REALTIME, GATING, TRACKING, PHASE_ORDERING, PHASE_RESCANNING, RETROSPECTIVE, CORRECTION
Respiratory Signal Source	Recommended	5200,9229 > 0018,9171	Signal source from which respiratory motion is derived.<br>Defined Terms: NONE, BELT, NASAL_PROBE, CO2_SENSOR, NAVIGATOR, MR_PHASE, ECG
Respiratory Trigger Delay Threshold	Recommended	5200,9229 > 0020,9256	Respiratory trigger threshold in percent of the chest expansion for the frame relative to the last Respiratory-Peak.

Respiratory Trigger Type	Recommended	5200,9229 > 0020,9250	Characteristic of the respiratory signal used to the define the respiratory triggering.<br>Defined Terms: TIME, AMPLITUDE, BOTH
Enhanced MR Image Functional Group Macros: MR FOV/Geometry			
MR Acquisition Frequency Encoding Steps	Essential	5200,9229 > 0018,9125 > 0018,9058	Number of Frequency Encoding steps (kx) acquired
MR Acquisition Phase Encoding Steps in-plane	Essential	5200,9229 > 0018,9125 > 0018,9231	Number of In-Plane Phase Encoding steps (ky) acquired
MR Acquisition Phase Encoding Steps out-of-plane	Essential	5200,9229 > 0018,9125 > 0018,9232	Number of Out-of-Plane Phase Encoding steps (kz) acquired
Enhanced MR Image Functional Group Macros: MR Modifier			
Inversion Recovery	Essential	5200,9229 > 0018,9115 > 0018,9009	Inversion Recovery preparatory sequence.<br>Enumerated Values: YES, NO
Inversion Times	Essential	5200,9229 > 0018,9115 > 0018,9079	Times in ms after the middle of inverting RF pulse to middle of excitation pulse to detect the amount of longitudinal magnetization.
Flow Compensation	Essential	5200,9229 > 0018,9115 > 0018,9010	Flow Compensation.<br>Defined Terms: ACCELERATION, VELOCITY, OTHER, NONE
Flow Compensation Direction	Essential	5200,9229 > 0018,9115 > 0018,9183	Flow Compensation Direction.<br>Enumerated Values: PHASE, FREQUENCY, SLICE_SELECT, SLICE_AND_FREQ, SLICE_FREQ_PHASE, PHASE_AND_FREQ, SLICE_AND_PHASE, OTHER
Spoiling	Essential	5200,9229 > 0018,9115 > 0018,9016	Spoiling.<br>Enumerated Values: RF, GRADIENT, RF_AND_GRADIENT, NONE
T2 Preparation	Essential	5200,9229 > 0018,9115 > 0018,9021	T2 prepared Pulse Sequence.<br>Enumerated Values: YES, NO

Spectrally Selected Excitation	Essential	5200,9229 > 0018,9115 > 0018,9026	Spectrally Selected Excitation.<br>Enumerated Values: WATER, FAT, NONE
Spatial Pre-saturation	Essential	5200,9229 > 0018,9115 > 0018,9027	Spatial Pre-saturation.<br>Defined Terms: SLAB, NONE
Partial Fourier	Essential	5200,9229 > 0018,9115 > 0018,9081	Partial Fourier.<br>Enumerated Values: YES, NO
Partial Fourier Direction	Recommended	5200,9229 > 0018,9115 > 0018,9036	Direction of Partial Fourier.<br>Enumerated Values: PHASE, FREQUENCY, SLICE_SELECT, COMBINATION
Parallel Acquisition	Essential	5200,9229 > 0018,9115 > 0018,9077	Parallel acquisition has been used to reduce measurement time.<br>Enumerated Values: YES, NO
Parallel Acquisition Technique	Essential	5200,9229 > 0018,9115 > 0018,9078	Parallel acquisition characteristics.<br>Defined Terms: PILS, SENSE, SMASH, OTHER
Parallel Reduction Factor In-plane	Essential	5200,9229 > 0018,9115 > 0018,9069	Measurement time reduction factor expressed as ratio of original and reduced measurement time for the in-plane direction.
Parallel Reduction Factor out-of-plane	Recommended	5200,9229 > 0018,9115 > 0018,9155	Measurement time reduction factor expressed as ratio of original and reduced measurement time for the out-of-plane direction
Parallel Reduction Factor Second In-plane	Recommended	5200,9229 > 0018,9115 > 0018,9168	Measurement time reduction factor expressed as ratio of original and reduced measurement time for the second in-plane direction.<br>Only required for MR Spectroscopy SOP Instances.
Enhanced MR Image Functional Group Macros: MR Imaging Modifier			
Magnetization Transfer	Essential	5200,9229 > 0018,9006 > 0018,9020	Magnetization Transfer pulse sequence.<br>Enumerated Values: ON_RESONANCE, OFF_RESONANCE, NONE
Enhanced MR Image Functional Group Macros: MR Spatial Saturation			

Slab Thickness	Essential	5200,9229 ><br>0018,9107 ><br>0018,9104	Thickness of slab in mm.
Slab Orientation	Essential	5200,9229 ><br>0018,9107 ><br>0018,9105	The direction cosines of a normal vector perpendicular to the saturation plane with respect to the patient.
Mid Slab Position	Essential	5200,9229 ><br>0018,9107 ><br>0018,9106	The x, y, and z coordinates of the midpoint of the slab plane in mm with respect to the patient.
Enhanced MR Image Functional Group Macros: MR Velocity Encoding			
Velocity Encoding Direction	Recommended	5200,9229 ><br>0018,9197 ><br>0018,9090	Velocity encoding directions used for acquisition.<br>The direction cosines of the velocity encoding vector with respect to the patient. See Section C.7.6.2.1.1 for further explanation.
MR Pulse Sequence Module (Enhanced MR Image)			
Pulse Sequence Name	Essential	0018,9005	Name of the pulse sequence for annotation purposes. Potentially vendor-specific name.
MR Acquisition Type	Essential	0018,0023	Identification of spatial data encoding scheme.<br>Defined Terms: 1D, 2D, 3D
Echo Pulse Sequence	Essential	0018,9008	Echo category of pulse sequences.<br>Enumerated Values: SPIN, GRADIENT, BOTH
Multiple Spin Echo	Essential	0018,9011	Multiple Spin Echo category of pulse sequence used to collect different lines in k-space for a single frame.<br>Enumerated Values: YES, NO
Multi-planar Excitation	Essential	0018,9012	Technique that simultaneously excites several volumes.<br>Enumerated Values: YES, NO
Phase Contrast	Essential	0018,9014	Phase contrast pulse sequence is a pulse sequence in which the flowing spins are velocity encoded in phase.<br>Enumerated Values: YES, NO
Time of Flight Contrast	Recommended	0018,9015	Time of Flight contrast is created by the inflow of blood in the saturated plane.

			Enumerated Values: YES, NO
Arterial Spin Labeling Contrast	Essential	0018,9250	Arterial Spin Labeling contrast technique.<br>Enumerated Values: CONTINUOUS (a single long low powered RF pulse), PSEUDOCONTINUOUS (multiple short low powered RF pulses), PULSED (a single short high powered RF pulse)
Steady State Pulse Sequence	Essential	0018,9017	Steady State Sequence.<br>Defined Terms: FREE_PRECESSION, TRANSVERSE, TIME_REVERSED, LONGITUDINAL, NONE
Echo Planar Pulse Sequence	Essential	0018,9018	Echo Planar category of Pulse Sequences.<br>Enumerated Values: YES, NO
Saturation Recovery	Essential	0018,9024	Saturation recovery pulse sequence.<br>Enumerated Values: YES, NO
Spectrally Selected Suppression	Essential	0018,9025	Spectrally Selected Suppression.<br>Defined Terms: FAT, WATER, FAT_AND_WATER, SILICON_GEL, NONE
Oversampling Phase	Recommended	0018,9029	Oversampling Phase.<br>Enumerated Values: 2D (phase direction), 3D (out of plane direction), 2D_3D (both), NONE
Geometry of k-Space Traversal	Essential	0018,9032	Geometry category of k-Space traversal.<br>Defined Terms: RECTILINEAR, RADIAL, SPIRAL
Rectilinear Phase Encode Reordering	Recommended	0018,9034	Rectilinear phase encode reordering.<br>Defined Terms: LINEAR, CENTRIC, SEGMENTED, REVERSE_LINEAR, REVERSE_CENTRIC
Segmented k-Space Traversal	Recommended	0018,9033	Segmented k-Space traversal. If Geometry of k-Space Traversal is rectilinear, multiple lines can be acquired at one time. If Geometry of k-Space Traversal is spiral or radial, paths can be interleaved and acquired at one time.

			Enumerated Values: SINGLE (successive single echo coverage), PARTIAL (segmented coverage), FULL (single shot full coverage)
Coverage of k-Space	Recommended	0018,9094	Coverage of k-Space in the ky-kz plane.<br>Defined Terms: FULL, CYLINDRICAL, ELLIPSOIDAL, WEIGHTED
Number of k-Space Trajectories	Recommended	0018,9093	Number of interleaves or shots.

Table S4: Summary results for CT Imaging category

**Element**	**Assessment**	**DICOM Tag**	**Definition**
General Reference Module			
Derivation Description	Essential	0008,2111	A text description of how this image was derived.
CT Image Module			
Image Type	Recommended	0008,0008	*For CT data, the 3rd value has these defined terms: AXIAL, LOCALIZER.*<br>*The 4th value is required if Multi-energy Acquisition (0018,9361) has a value of YES.*
Multi-energy CT Acquisition	Essential	0018,9361	Indicates whether the image is created by means of Multi-energy technique.<br>Enumerated Values: YES, NO
Rescale Intercept	Essential	0028,1052	The value b in relationship between stored values (SV) and the output units.<br>Output units = m*SV+b<br>If Image Type (0008,0008) Value 1 is ORIGINAL and Value 3 is not LOCALIZER, and Multi-energy CT Acquisition (0018,9361) is either absent or NO, output units shall be Hounsfield Units (HU).
Rescale Slope	Essential	0028,1053	The slope (m) in the equation specified in Rescale Intercept (0028,1052).

Rescale Type	Essential	0028,1054	Specifies the output units of Rescale Slope (0028,1053) and Rescale Intercept (0028,1052).<br>See Section C.11.1.1.2 for Defined Terms and further explanation.<br>Required if the Rescale Type is not HU (Hounsfield Units), or Multi-energy CT Acquisition (0018,9361) is YES. May be present otherwise.
KVP	Essential	0018,0060	Peak kilo voltage output of the X-Ray generator used.
Acquisition Number	Essential	0020,0012	A number identifying the single continuous gathering of data over a period of time that resulted in this image.
Scan Options	Essential	0018,0022	Parameters of scanning sequence.
Data Collection Center (Patient)	Recommended	0018,9313	The x, y, and z coordinates (in the Patient-Based Coordinate System) in mm of the center of the region in which data were collected. See Section C.8.15.3.6.1.
Distance Source to Detector	Recommended	0018,1110	Distance in mm from source to detector center.
Distance Source to Patient	Recommended	0018,1111	Distance in mm from source to isocenter (center of field of view).
Magnification	Recommended	NA	*Computed from Distance Source to Detector (0018,1110) and Distance Source to Patient (0018,1111)*
Exposure Time	Essential	0018,1150	Time of X-Ray exposure in msec.<br>If Acquisition Type (0018,9302) equals SPIRAL, the value of this Attribute shall be Revolution Time (0018,9305) divided by the Spiral Pitch Factor (0018,9311). See Section C.8.15.3.8.1 and Section C.8.15.3.2.1.
X-Ray Tube Current	Essential	0018,1151	X-Ray Tube Current in mA.
Filter Type	Essential	0018,1160	Label for the type of filter inserted into the X-Ray beam.
Focal Spot(s)	Essential	0018,1190	Size of the focal spot in mm. For devices with variable focal spot or

			multiple focal spots, small dimension followed by large dimension.
Convolution Kernel	Essential	0018,1210	A label describing the convolution kernel or algorithm used to reconstruct the data.
Single Collimation Width	Recommended	0018,9306	The width of a single row of acquired data (in mm).
Total Collimation Width	Recommended	0018,9307	The width of the total collimation (in mm) over the area of active X-Ray detection.
Exposure Modulation Type	Essential	0018,9323	A label describing the type of exposure modulation used for the purpose of limiting the dose.<br>Defined Terms: NONE
Isocenter Position	Recommended	300A,012C	Isocenter coordinates (x,y,z), in mm. Specifies the location of the machine isocenter in the Patient-Based Coordinate System associated with the Frame of Reference. It allows transformation from the Equipment-Based Coordinate System to the Patient-Based Coordinate System.
Enhanced CT Image Functional Group Macros: CT Acquisition Type Macro			
Acquisition Mode	Recommended	5200,9229 ><br>0018,9301 ><br>0018,9302	Description of the method used during acquisition of this frame.<br>*Defined Terms: SEQUENCED, SPIRAL, CONSTANT_ANGLE, STATIONARY, FREE*
Respiratory Synchronization Module (Enhanced CT Image)			
Respiratory Motion Compensation Technique	Essential	0018,9170	Applied technique to reduce respiratory motion artifacts.<br>Defined Terms: NONE, BREATH_HOLD, REALTIME, GATING, TRACKING, PHASE_ORDERING, PHASE_RESCANNING, RETROSPECTIVE, CORRECTION
Private Elements			
Beam Hardening Correction Flag	Essential	NA	A flag used to define whether the projection data has been corrected for beam hardening effects*

*McCollough, et. al. have suggested a set of private DICOM tags to record vendor neutral data in raw projection data. One of the private elements is Beam Hardening Correction Flag with values of "YES" or "NO".

References

McCollough, C.H., Chen, B., Holmes, D., III, Duan, X., Yu, Z., Yu, L., Leng, S., Fletcher, J. (2020). Data from Low Dose CT Image and Projection Data [Data set]. The Cancer Imaging Archive. https://wiki.cancerimagingarchive.net/download/attachments/52758026/DICOM-CT-PD%20User%20Manual_Version%203.pdf?version=1&modificationDate=1586187875073&api=v2
